# The impact of adjuvant chemotherapy in older breast cancer patients on clinical and biological aging parameters

**DOI:** 10.18632/oncotarget.8796

**Published:** 2016-04-18

**Authors:** Barbara Brouwers, Sigrid Hatse, Lissandra Dal Lago, Patrick Neven, Peter Vuylsteke, Bruna Dalmasso, Guy Debrock, Heidi Van Den Bulck, Ann Smeets, Oliver Bechter, Jithendra Kini Bailur, Cindy Kenis, Annouschka Laenen, Patrick Schöffski, Graham Pawelec, Fabrice Journe, Ghanem-Elias Ghanem, Hans Wildiers

**Affiliations:** ^1^ Laboratory of Experimental Oncology (LEO), Department of Oncology, KU Leuven, and Department of General Medical Oncology, University Hospitals Leuven, Leuven Cancer Institute, Leuven, Belgium; ^2^ Department of Medicine, Institut Jules Bordet, Université Libre de Bruxelles, Brussels, Belgium; ^3^ Multidisciplinary Breast Center, University Hospitals Leuven, Leuven, Belgium; ^4^ Department of Medical Oncology, Clinique et Maternité Sainte-Elisabeth, Namur, Belgium; ^5^ Department of Internal Medicine, Istituto di Ricerca a Carattere Clinico e Scientifico (IRCCS), Azienda Ospedaliera Universitaria (AOU) San Martino Istituto Nazionale Tumori (IST), Genoa, Italy; ^6^ Department of Medical Oncology, Ziekenhuizen Oost Limburg (ZOL), Genk, Belgium; ^7^ Department of Medical Oncology, Imelda Ziekenhuis Bondheiden, Belgium; ^8^ Department of Internal Medicine II, Centre for Medical Research, University of Tübingen, Tübingen, Germany; ^9^ Department of General Medical Oncology and Geriatric Medicine, University Hospitals Leuven, Belgium; ^10^ Interuniversity Centre for Biostatistics and Statistical Bioinformatics, Leuven, Belgium; ^11^ School of Science and Technology, Nottingham Trent University, Nottingham, UK; ^12^ Laboratory of Oncology and Experimental Surgery, Institut Jules Bordet, Université Libre de Bruxelles, Brussels, Belgium

**Keywords:** breast cancer, older patients, adjuvant chemotherapy, biological aging, aging biomarkers

## Abstract

**Purpose:**

This prospective observational study aimed to evaluate the impact of adjuvant chemotherapy on biological and clinical markers of aging and frailty.

**Methods:**

Women ≥ 70 years old with early breast cancer were enrolled after surgery and assigned to a chemotherapy (Docetaxel and Cyclophosphamide) group (CTG, *n*=57) or control group (CG, *n*=52) depending on their planned adjuvant treatment. Full geriatric assessment (GA) and Quality of Life (QoL) were evaluated at inclusion (T0), after 3 months (T1) and at 1 year (T2). Blood samples were collected to measure leukocyte telomere length (LTL), levels of interleukin-6 (IL-6) and other circulating markers potentially informative for aging and frailty: Interleukin-10 (IL-10), Tumor Necrosis Factor Alpha (TNF-α), Insulin-like Growth Factor 1 (IGF-1), Monocyte Chemotactic Protein 1 (MCP-1) and Regulated on Activation, Normal T cell Expressed and Secreted (RANTES).

**Results:**

LTL decreased significantly but comparably in both groups, whereas IL-6 was unchanged at T2. However, IL-10, TNF-α, IGF-1 and MCP-1 suggested a minor biological aging effect of chemotherapy. Clinical frailty and QoL decreased at T1 in the CTG, but recovered at T2, while remaining stable in the CG.

**Conclusion:**

Chemotherapy (TC) is unlikely to amplify clinical aging or induce frailty at 1 year. Accordingly, there is no impact on the most established aging biomarkers (LTL, IL-6).

## INTRODUCTION

The incidence of breast cancer, the most frequent tumor occurring in women, increases with age. While adequate treatment can improve outcome and survival in the elderly, concerns over side effects or the idea of futility result in a lower use of adjuvant chemotherapy in this patient population. This might be one of the reasons why cancer-related mortality is higher in older patients [1]. The high variability of individual health status constitutes a major challenge in offering optimal therapy to the elderly. A comprehensive geriatric assessment (GA), evaluating functional status, comorbidity, socio-economic condition, nutrition and polypharmacy, is therefore necessary, and has been recommended by the International Society of Geriatric Oncology (SIOG) [2]. Based on our own findings, biological markers of aging and frailty could add on to this clinical evaluation [3, 4].

In line with the complexity of the aging process, a huge variety of potential aging biomarkers has been described. A crucial role has been attributed to telomeres, in cells and tissues subjected to replicative aging. They are incompletely replicated in somatic cells and shorten with each cellular division. Therefore, leukocyte telomere length (LTL) can serve as a marker of a cell's replicative “age” [5], and, in extension, can mirror a person's biological age [6]. LTL correlates with several aging-related syndromes [7]. An increasing low-grade chronic inflammatory status, reflected by an altered plasma level of multiple inflammatory mediators [8–10], is another hallmark of aging. Levels of interleukin-6 (IL-6) and tumor necrosis factor alpha (TNF-α) continuously rise with age, and have been associated with several aging-related syndromes [11–13]. Conversely, the anti-inflammatory cytokine interleukin-10 (IL-10) tends to decrease in blood during aging [14] and age-related diseases [15]. Furthermore, several chemokines also change during aging [16–19] : Monocyte Chemotactic protein 1 (MCP-1) blood levels are higher in older people compared to younger individuals [20–22]. Regulated on Activation, Normal T cell Expressed and Secreted (RANTES), has shown to undergo age-related changes as well, although, results from the literature are not consistent [21, 22]. Additionally, perturbation of the insulin/insulin-like growth factor 1 (IGF-1) metabolic pathway has been implicated in aging-related disease, and reduced longevity in both animal models [23–25] and humans [26, 27, 12].

Chemotherapy may influence the aging process via a variety of different mechanisms. Firstly, anticancer agents can induce cellular senescence through DNA damage [28], either directly or indirectly via generation of free radical intermediates and inhibition of DNA repair enzymes. Secondly, chemotherapy may specifically accelerate telomere attrition in leukocytes, most likely due to direct telomere damage or possibly by inhibition of the enzyme telomerase [29]. Repeated cycles of intense hematological repopulation during chemotherapy may shorten telomeres more rapidly if telomerase is not compensating for endochromosomal DNA loss [30–32]. Such effects of anticancer drugs on the replicative capacity of blood cells may be more pronounced in older compared to younger patients [33]. Finally, neuroendocrine and immune functions can also be affected by chemotherapy and by corticosteroids that are often incorporated in chemotherapeutic regimens [34]. Chemotherapy might thus be expected to accelerate aging [35, 36, 33, 37, 38]. It has been hypothesized that an increased rate of molecular aging might explain some of the delayed adverse events linked to chemotherapy [39]. However, long-term follow-up data, on both clinical and biological repercussions of chemotherapeutic treatments, have never been reported

To ensure optimal treatment decisions in older patients, it is of utmost importance to further elucidate the impact of chemotherapy on the aging process, not only biologically, but most particularly in terms of clinical repercussion. Here, we report a prospective study to assess the effect of chemotherapy on biological and clinical aging markers in older patients with breast cancer.

## RESULTS

In total, 109 consecutive subjects were enrolled in the study: 57 in the chemotherapy group (CTG) and 52 in the control group (CG). Almost all CTG patients completed their adjuvant chemotherapy. One patient stopped after the first cycle, one after the second cycle and two patients after the third cycle because of adverse events (allergy, severe infection and overall intolerance). Two other patients stopped after 1 cycle because of an allergic reaction, but resumed chemotherapy with a taxane-free, anthracyclin containing regimen. Baseline tumor and treatment characteristics are described in Table 1.

**Table 1:** Baseline patient and tumor characteristics

	Chemo Group (*n* = 57)	Control Group (*n* = 52)
Age Median, years (range)	73.5 (70-80)	75.0 (70-90)
pT 1 2 3 4	n(%) 11(19) 37 (65) 6 (11) 3 (5)	n(%) 21 (40) 30 (58) 0 (0) 1 (2)
pN 0 1-3	n (%) 18 (33) 36 (67)	n (%) 27 (53) 24 (47)
Breast cancer phenotype^§^ Basal like HER2 positive (ER negative) Luminal A Luminal B HER2 negative Luminal B HER2 positive	n (%) 11 (19) 6 (10) 9 (16) 22 (39) 9 (16)	n (%) 0 (0) 0 (0) 35 (67) 16 (31) 1 (2)
Adjuvant therapy TC chemotherapy G-CSF primary prophylaxis Trastuzumab 1 year Endocrine therapy Radiotherapy	n (%) 56 (100) 48 (86) 15 (27) 40 (71) 46 (82)	n (%) 0 (0) 0 (0) 0 (0) 52 (100) 32 (62)

Abbreviations : ER : Estrogen Receptor; TC : Docetaxel-Cyclophosphamide; G-CSF : Granulocyte-Colony Stimulating Factor

§ :Breast cancer phenotype : see ref 47 in manuscript, Goldhirsh et al.

**Figure 1:**
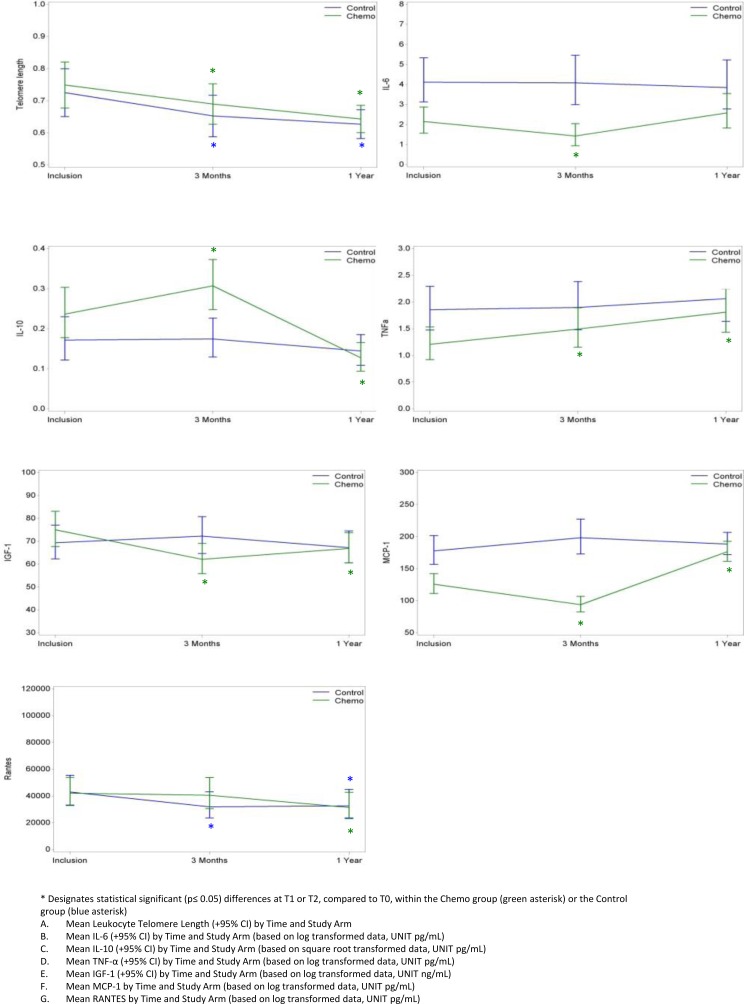
Evolution over time of aging biomarker results in the Chemo and Control Groups * Designates statistical significant (p≤ 0.05) differences at T1 or T2, compared to T0, within the Chemo group (green asterisk) or the Control group (blue asterisk) **A.** Mean Leukocyte Telomere Length (+95% CI) by Time and Study Arm **B.** Mean IL-6 (+95% CI) by Time and Study Arm (based on log transformed data, UNIT pg/mL) **C.** Mean IL-10 (+95% CI) by Time and Study Arm (based on square root transformed data, UNIT pg/mL) **D.** Mean TNF-α (+95% CI) by Time and Study Arm (based on log transformed data, UNIT pg/mL) **E.** Mean IGF-1 (+95% CI) by Time and Study Arm (based on log transformed data, UNIT ng/mL) **F.** Mean MCP-1 by Time and Study Arm (based on log transformed data, UNIT pg/mL) **G.** Mean RANTES by Time and Study Arm (based on log transformed data, UNIT pg/mL).

Results of the different biomarker assays at the 3 time points (T0, inclusion; T1, at 3 months; T2, at 1 year) and their evolution over time are shown in Table 2 and Figure 1. In brief, LTL was similar in both cohorts at inclusion, and decreased to the same extent in both groups, indicating no difference in evolution in the two cohorts (test for interaction p=0.88). Also for RANTES, the evolution was similar in both groups. In contrast, the other 5 biomarkers remained stable in the CG while significantly changing in the CTG. IL-6 decreased at T1 and returned to initial levels at T2; MCP-1 decreased at T1 but increased above baseline value at T2; IGF-1 showed a similar initial decline at T1 but only slightly recovered at T2. On the other hand, IL-10 increased at T1 but decreased at T2 and TNF-α levels were increased at both T1 and T2. To determine if differences in baseline frailty between groups could have influenced these results, we repeated the time interaction analysis correcting for frailty at T0. This analysis showed similar results (Table 2).

**Table 2:** Aging biomarker results at baseline (T0), 3 months (T1), and 1 year (T2), and their differential evolution over time in Chemo and Control Groups

	Chemo Group (n=57)			Evolution Over Time Chemo Group		Control Group(n=52)			Evolution Over Time Control Group		Differential Evolution Chemo and Control (TimeInteraction)
	T0	T1	T2	T0 → T1	T0 → T2	T0	T1	T2	T0 → T1	T0 → T2	
LTL N T/S mean +/− SD	45 0.7 +/− 0.2	46 0.7 +/− 0.3	49 0.6 +/− 0.2	**p=0.05** ***p* =0.05**	**p<0.01** ***p* <0.01**	41 0.7 +/−0.3	45 0.6 +/−0.15	44 0.6 +/−0.14	**p=0.02** ***p* =0.02**	**p<0.01** ***p* <0.01**	p=0.88 *p*=0.87
IL-6 N pg/mL mean +/− SD	56 3.2 +/− 3.7	55 2.3 +/− 3.7	51 4.5 +/− 9.2	**p=0.02** ***p* =0.02**	p=0.27 ***p* =0.26**	52 7.0 +/−13.9	48 11.4 +/−38.5	46 5.6 +/−6.1	p=0.95 *p*=0.77	p=0.66 *p*=0.45	**p<0.01** ***p* <0.04**
IL-10 N pg/mL mean +/− SD	51 0.3+/− 0.4	50 0.3 +/− 0.3	47 0.2 +/− 0.1	**p=0.05** ***p* =0.01**	**p<0.01** ***p* <0.01**	50 0.2 +/−0.2	47 0.2 +/−0.2	46 0.2 +/−0.1	p=0.92 *p*=0.96	p=0.28 *p*=0.23	**p<0.01** ***p* <0.01**
TNF-alpha N pg/mL mean +/− SD	56 2.5 +/− 10.1	55 2.9 +/− 9.7	51 3.3 +/− 9.6	**p<0.01** ***p* <0.01**	**p<0.01** ***p* <0.01**	52 2.3 +/−3.1	48 2.5 +/−3.4	46 2.5 +/−3.1	p=0.71 *p*=0.71	p=0.08 *p*=0.06	**p<0.01** ***p* =0.01**
MCP-1 N pg/mL mean +/− SD	55 143 +/− 70	55 110.7 +/− 70	51 183.2+/− 48	**p<0.01** ***p* <0.01**	**p<0.01** ***p* <0.01**	52 189 +/−78	48 219 +/−131	46 207 +/−108	p=0.14 *p*=0.16	p=0.34 *p*=0.29	**p<0.01** ***p* <0.01**
Rantes N pg/mL mean +/− SD	55 59562+/− 46691	55 61411+/− 53735	51 51903+/− 47600	p=0.78 *p*=0.83	**p=0.01** ***p* =0.01**	52 59004 +/−43436	48 53215 +/−46461	46 55421 +/−48231	**p=0.03** ***p* =0.03**	**p=0.03** *p*=0.03	p=0.29 *p*=0.28
IGF-1 N ng/mL mean +/− SD	55 79 +/− 26	54 67 +/− 26	51 70 +/− 24	**p<0.01** ***p* <0.01**	**p<0.01** ***p* <0.01**	51 76 +/−36	48 77 +/−27	46 74 +/−34	p=0.31 *p*=0.35	p=0.48 *p*=0.64	**p<0.01** ***p* <0.01**

Abbreviations. SD: Standard Deviation; CI: Confidence Interval; LTL: Leukocyte Telomere Length; IL-6: Interleukin-6; IL-10: Interleukin-10; TNF-alpha: Tumor Necrosis Factor Alpha; MCP-1: Monocyte Chemotactic Protein 1; RANTES: Regulated Upon Activation, Normal T cell Expressed and presumably Secreted; IGF-1: Insulin Like Growth Factor 1

**Figure 2:**
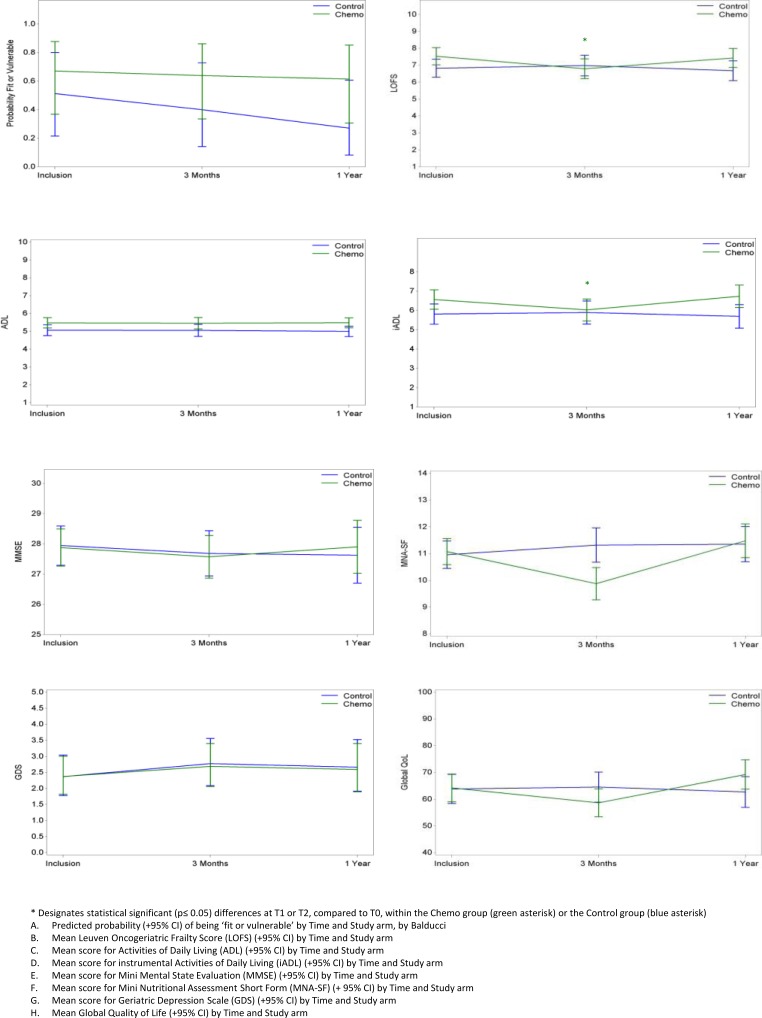
Evolution over time of geriatric assessment parameters in the Chemo and Control Groups * Designates statistical significant (p≤ 0.05) differences at T1 or T2, compared to T0, within the Chemo group (green asterisk) or the Control group (blue asterisk) **A.** Predicted probability (+95% CI) of being ‘fit or vulnerable’ by Time and Study arm, by Balducci **B.** Mean Leuven Oncogeriatric Frailty Score (LOFS) (+95% CI) by Time and Study arm **C.** Mean score for Activities of Daily Living (ADL) (+95% CI) by Time and Study arm **D.** Mean score for instrumental Activities of Daily Living (iADL) (+95% CI) by Time and Study arm **E.** Mean score for Mini Mental State Evaluation (MMSE) (+95% CI) by Time and Study arm **F.** Mean score for Mini Nutritional Assessment Short Form (MNA-SF) (+ 95% CI) by Time and Study arm **G.** Mean score for Geriatric Depression Scale (GDS) (+95% CI) by Time and Study arm **H.** Mean Global Quality of Life (+95% CI) by Time and Study arm

For background on geriatric assessment and our newly developed frailty score the ‘Leuven Oncogeriatric Frailty Score (LOFS)’, we refer to the section patients and methods and appendix 1. GA results at the 3 time points, and the differential evolution over time (with and without correction for frailty) are displayed in Table 3 and Figure 2. A significant interaction test, pointing to a differential evolution in time between both groups, was found for LOFS, instrumental activities of daily living (iADL), Mini Nutritrional Assessment – short form (MNA-SF) and Global Quality of Life (Global QoL), while this test was not significant for Activities of Daily Living (ADL), Mini Mental Status Evaluation (MMSE), Geriatric Depression Scale - 15 (GDS-15) and Charlson Comorbidity Index (CCI). A marked decline in LOFS, iADL, MNA-SF and Global QoL was noted at T1 in the CTG but not CG. However, all significant differences noted at T1 in the CTG returned to normal at T2. No significant modifications of frailty level according to Balducci were found in either of the two groups: the odds ratio for being fit rather than vulnerable, or vulnerable rather than frail according to this index was 0.90 (95% CI 0.27-3.07) from T0 to T1 and 0.63 (95% CI 0.21-1.90) from T0 to T2 in the CTG, and there was no difference with the CG (test for interaction p=0.63) (see Figure 2A).

**Table 3:** Geriatric assessment results at baseline (T0), 3 months (T1), and 1 year (T2), and their differential evolution over time in Chemo and Control Groups

	Chemo Group (n=57)			Evolution Over Time Chemo Group		Control Group (n=52)			Evolution Over Time Control Group		Differential Evolution (Time Interaction)
	T0	T1	T2	T0 → T1	T0 → T2	T0	T1	T2	T0 → T1	T0 → T2	
Frailty(Balducci) N Fit n (%) Vulnerable n (%) Frail n (%)	56 12 (21) 21 (35) 23 (41)	53 8 (15) 23 (43) 22 (42)	48 10 (21) 15 (31) 23 (48)	p=0.87	p=0.41	52 10 (19) 17 (33) 25 (48)	48 9 (19) 13 (27) 26 (54)	46 7 (15) 13 (28) 26 (57)	p=0.34	p=0.77	*p*=0.63α
LOFS N Mean +/− SD	56 7.5 +/− 2	53 6.7 +/− 2	48 7.4 +/− 2	**p<0.01**	*p*=0.60	51 6.8 +/− 2	48 7.0 +/− 2	46 6.8 +/− 2	p=0.48	p=0.45	**p<0.01**
ADL N Mean +/− SD 6§ n (%) 0-5 n (%)	56 5.5 +/− 1 33 (59) 23 (41)	56 5.4 +/− 1 35 (62) 21 (37)	51 5.5 +/− 1 30 (59) 21 (41)	p=0.88 *p*=0.88	p=0.96 *p*=0.99	52 5.1 +/− 1 29 (56) 23 (44)	48 5.0 +/− 1 23 (48) 25 (52)	46 5.0 +/− 1 21 (46) 25 (54)	p=0.94 *p*=0.85	p=0.57 *p*=0.47	p=0.77 *p*=0.76
IADL N Mean +/− SD 8§ n (%) 0-7 n (%)	57 6.6 +/− 2 24 (42) 33 (58)	56 6.0 +/− 2 16 (29) 40 (71)	51 6.8 +/− 2 26 (51) 25 (49)	**p<0.01** *p*=0.01	p=0.39 *p*=0.39	52 5.8 +/− 2 17 (33) 35 (67)	48 5.8 +/− 2 17 (35) 31 (65)	46 5.7 +/− 3 17 (37) 29 (63)	p=0.71 *p*=0.75	p=0.57 *p*=0.60	**p<0.01** *p*=0.01
Previous falls N No n (%) Yes n (%)	57 44 (77) 13 (23)	N/A	50 34 (68) 16 (32)	N/A	p=0.28 *p*=0.25	52 28 (54) 24 (46)	N/A	46 29 (63) 17 (37)	N/A	p=0.33 *p*=0.37	p=0.15β *p*=0.15 β
MMSE N Mean +/− SD	57 27.9 +/− 2	56 27.6 +/− 3	51 27.9 +/− 3	p=0.22 *p*=0.19	p=0.94 *p*=0.96	52 27.9 +/− 2	48 27.8 +/− 3	46 27.9 +/− 4	p=0.34 *p*=0.31	p=0.40 *p*=0.41	p=0.77 *p*=0.78
GDS-15 N Mean +/− SD 0-4§ n (%) 5-15 n (%)	55 2.9 +/− 2 46 (84) 9 (16)	56 3.1 +/− 2 43 (77) 13 (23)	50 3.1 +/− 3 38 (76) 12 (24)	p=0.31 *p*=0.23	p=0.50 *p*=0.51	46 2.8 +/− 2 35 (76) 11 (24)	47 3.5 +/− 3 32 (68) 15 (32)	45 3.1 +/− 3 35 (78) 10 (22)	p=0.23 *p*=0.25	p=0.40 *p*=0.40	p=0.98 *p*=0.98
MNA-SF N Mean +/− SD ≥ 12§ n (%) < 11 n (%)	57 11.1 +/− 2 25 (44) 32 (56)	55 9.9 +/− 2 16 (29) 39 (71)	50 11.5 +/− 2 27 (54) 23 (46)	**p<0.01** *p*<0.01	p=0.19 *p*=0.13	51 11.0 +/− 2 23 (45) 28 (55)	48 11.3 +/− 2 29 (60) 19 (40)	46 11.4 +/− 2 23 (50) 23 (50)	p=0.24 *p*=0.32	p=0.23 *p*=0.21	**p<0.01** *p*<0.01
CCI N Mean +/− SD 0§ n (%) 1 n (%) >=2 n (%)	54 0.6 +/− 1 32 (59) 12 (22) 10 (19)	53 0.7 +/− 1 32 (60) 10 (19) 11 (21)	49 0.7 +/− 1 28 (57) 12 (25) 9 (18)	p=0.42 *p*=0.48	p=0.16 *p*=0.78	52 1.1 +/− 2 27 (52) 12 (23) 13 (25)	50 1.1 +/− 2 27 (54) 10 (20) 13 (26)	46 1.2 +/− 2 22 (48) 11 (24) 13 (28)	p=0.86 *p*=0.97	p=0.35 *p*=0.91	p=0.63θ *p*=0.86 θ
G8 N Mean +/− SD >14 n (%) ≤ 14 n (%)	56 14.2 +/− 2 28 (50) 28 (50)	NA	NA	N/A	N/A	52 13.7 +/− 2 22 (42) 30 (58)	NA	NA	N/A	N/A	N/A
Global QoL N Mean +/− SD	57 64.2 +/− 17	56 58.5 +/− 20	50 69.5 +/− 22	p=0.06 *p*=0.05	p=0.11 *p*=0.08	52 63.8 +/− 17	48 64.6 +/− 20	46 63.6 +/− 23	p=0.83 *p*=0.83	p=0.73 *p*=0.96	**p=0.02** *p*=0.03

Abbreviations. SD: Standard Deviation; CI: Confidence Interval; ADL: Activities of Daily Living; IADL: Instrumental Activities of Daily Living; MMSE: Mini Mental State Examination; GDS: Geriatric Depression Scale; MNA-SF: Mini Nutritional Assessment-Short Form; MNA: Mini Nutritional Assessment

§: Maximum score, no abnormalities

α: calculation based on probability of being fit or vulnerable by time and study arm

β: calculation based on probability of falling by time and study arm

θ: calculation based on probability of having the lowest score at CCI, by time and study arm

p-values in italic font show results corrected for patient frailty level at baseline

Within the CTG we explored the influence of baseline frailty on the time evolution of biological and clinical aging markers. Because the very small number of truly frail patients in this chemotherapy group, we chose to dichotomise the patients comparing fit patients to vulnerable+frail patients according to Balducci, and patients with LOFS ≥ 8 to patients with LOFS < 8). Except for LTL evolution, that showed a significant time interaction with frailty status (p=0.04 for Balducci dichotomization and p=0.01 for LOFS dichotomization), no differences in evolution according to frailty status at the start were seen for other biomarkers. As for the clinical aging parameters, the evolution over time according to baseline frailty status showed to be different for MNA and Global Health (significant time interaction with Balducci category; p=0.02 and p=0.01 respectively) and for GDS and Falls (significant time interaction with LOFS category; p=0.04 and p=0.01 respectively), but not for CCI, ADL, iADL and MMSE.

Correlations of baseline (T0) aging biomarkers with chronological age and LOFS are shown in Table 4. LTL showed a significant correlation with LOFS but not with chronological age. Of all biomarkers, IL-6 was most strongly associated with both chronological age and LOFS: the higher IL-6, the higher chronological age and the lower the LOFS. TNFα showed a strong and highly significant positive correlation with chronological age. Associations with other aging biomarkers were not significant.

**Table 4:** Correlation of baseline aging biomarkers with chronological age and clinical aging (according to LOFS)

	Chronological age (years)			Clinical aging (LOFS)		
	Spearman correlation	*p*-value	*N*	Spearman correlation	*p*-value	*N*
Telomere length	−0.11	0.32	86	−0.27	0.01	85
IL-6	0.32	<0.01	108	−0.21	0.03	106
IL-10	−0.03	0.78	101	−0.05	0.62	99
IGF-1	−0.01	0.33	106	−0.03	0.75	104
TNF-α	0.34	<0.01	108	−0.18	0.06	106
MCP-1/CCL-2	0.18	0.07	107	−0.14	0.16	105
RANTES/CCL-5	−0.01	0.88	107	0.16	0.10	105

Abbreviations. LOFS : Leuven Oncogeriatric Frailty Score; IL-6 : Interleukin-6; IL-10 : Interleukin-10; IGF-1 : Insulin Like Growth Factor-1; TNF-α : Tumor Necrosis Factor-α; MCP-1/CCL2 : Monocyte chemotactic protein-1/Chemokine (C-C motif) ligand 2; RANTES/CCL5 : Regulated Upon Activation, Normal T cell Expressed and presumably Secreted/ Chemokine (C-C motif) Ligand 5

Adverse events occurring during the study period were recorded at 3 months and at one year, and are summarized in Table 5. As expected, toxicity was markedly more frequent in the CTG, particularly during treatment (i.e. between T0 and T1). We also assessed whether any of the aging biomarkers could predict the occurrence of grade II-III-IV toxicity at 3 months in the CTG. Analyses were performed for toxicity parameters that occurred in at least 5 patients. None of the aging biomarkers at baseline (T0) predicted development of grade II or higher toxicity, and neither did the Balducci score or LOFS (data not shown).

Unplanned readmissions occurred between 0 and 3 months in 12 patients (22%) of the CTG (N=54) and 3 patients (6%) of the CG (N=50). Between 3 and 12 months, these numbers were 9 (18%) for the CTG and 15 (32%) for the CG. However, none of the biomarkers at baseline (T0) nor Balducci or LOFS predicted an unplanned readmission during chemotherapy.

**Table 5:** Cumulative toxicity

Cumulative adverse event		Chemotherapy Group /Control group Grade in %			
		**1**	**2**	**3**	**4**
Febrile neutropenia	T0 **→** T1	0 / 0	0 / 0	13 / 0	0 / 0
	T1 → T2	0 / 0	0 / 0	0 / 0	0 / 0
Anemia	T0 → T1	63 / 18	11 / 8	2 / 0	0 / 0
	T1 → T2	18 / 25	2 / 4	0 / 0	0 / 0
Diarrhea	T0 → T1	26 / 2	4 / 0	2 / 0	0 / 0
	T1 → T2	2 / 0	2 / 0	0 / 0	0 / 0
Nausea/vomiting	T0 → T1	41 / 2	7 / 0	0 / 0	0 / 0
	T1 → T2	0 / 4	0 / 0	0 / 0	0 / 0
Anorexia	T0 → T1	46 / 6	9 / 0	2 / 0	0 / 0
	T1 → T2	6 / 2	6 / 4	0 / 0	0 / 0
Fatigue	T0 → T1	44 / 26	22 / 0	2 / 2	0 / 0
	T1 → T2	30 / 19	2 / 4	2 / 0	0 / 0
Pain	T0 → T1	30 / 28	9 / 2	0 / 0	0 / 0
	T1 → T2	40 / 53	4 / 2	0 / 0	0 / 0
Mucositis	T0 → T1	24 / 0	2 / 0	2 / 0	0 / 0
	T1 → T2	1 / 0	0 / 0	0 / 0	0 / 0
Sensory neuropathy	T0 → T1	15 / 4	0 / 0	0 / 0	0 / 0
	T1 → T2	8 / 0	0 / 0	0 / 2	0 / 0
Rash	T0 → T1	17 / 0	0 / 0	0 / 0	0 / 0
	T1 → T2	2 / 0	0 / 0	0 / 0	0 / 0

## DISCUSSION

Geriatric oncology is a growing discipline. Older breast cancer patients have a higher cancer-specific mortality [1], probably because therapy is withheld on concerns over side effects. Is this fear justified and are these suspected side effects actually related to the aging process? Some studies seem to show an accelerating effect of chemotherapy on the aging process [33, 35, 36, 38]. One could anticipate an increase in geriatric problems after chemotherapy. This could mislead the oncologist not to administer chemotherapy where it would otherwise have been indicated. However, data on this topic still remain disparate to date. DNA damage (and DNA damaging drugs) are suggested not necessarily to cause or accelerate aging [40], and no report exists that investigated alterations in aging biomarkers, attributed to chemotherapy, show an impact on clinical outcome. Therefore, we prospectively compared clinical and as well as potential biological aging markers in a cohort of older breast cancer patients given or not given chemotherapy after surgery. The patients were all >70 years of age, thus investigating a truly ‘older’ population. In those patients, geriatric assessment often reveals previously unknown age-related problems [2].

After 1 year of follow-up, we found that chemotherapy did not significantly influence established markers of clinical frailty. Our data only revealed a mild and transient decrease of global fitness status in older breast cancer patients undergoing chemotherapy: increased clinical frailty, as evidenced by a lower LOFS score, was noted in the CTG (but not CG) after 3 months of treatment, but the frailty status returned to baseline level after one year. Frailty status according to Balducci did not change during the time course of the study. Global QoL was also slightly decreased at 3 months in the CTG (but not the CG), but was also restored after 1 year. The temporary decrease in fitness and QoL is not unexpected, and can be explained by acute and subacute chemotherapy toxicity. However, our study has proven that overall, the TC regimen [41–43] (generally administered with primary G-CSF support) is well tolerated in older breast cancer patients. Apart from febrile neutropenia (13% grade III), we noticed few grade III, and even no grade IV side effects.

The absence of a pronounced aging/frailty-inducing effect of chemotherapy was further corroborated by measurements of some of the principal well-established aging biomarkers, such as LTL and IL-6. LTL was comparable in both groups at baseline and progressively decreased over the 1-year time course of the study with no significant difference between the two groups. This is in line with the well-known age-related process of progressive telomere attrition [6, 7, 44] but does not support the hypothesis that the aging process is accelerated by chemotherapy. Similar to LTL, the plasma marker IL-6 [13] did not reveal chemotherapy-induced aging progression when considering the evolution over the 1-year study period. On the other hand, several of the additional plasma biomarkers that have previously been associated with aging [13, 15, 21, 22, 25, 27, 45–47], did suggest a slight aging-promoting effect of chemotherapy: decreases in IL-10 and IGF-1 and increases in TNFα and MCP-1 from baseline to 1 year were significantly more pronounced in the CTG compared to the CG, suggesting accelerated biological aging. However, we tend to consider the clinical impact of alterations in only a few biomarkers that contribute to the so-called ‘inflammaging’ phenomenon rather minimal, especially as more robust aging biomarkers do not appear to show the same trend.

Although geriatric assessment parameters and the patient's perception of QoL did not change significantly at 1 year, one might argue that clinical changes may not immediately become visible, but might remain subclinical for a longer period of time. On the other hand, it was shown by Benitez-Beluga et al. [37] that biological changes induced by chemotherapy can recover to normal after a sufficiently long period of follow-up. Therefore, the transient changes observed shortly after treatment in our study, seem not very likely to have any clinical significance on the long term.

Of all evaluated biomarkers, IL-6 showed the strongest correlation with chronological age and LOFS, confirming its robustness as an aging biomarker as previously described [13]. Associations of the other aging biomarkers were less prominent and mostly not significant. It should be noted, though, that the cohort examined in this study only comprised elderly people within narrow age range (70 – 90 years). Hence, the lack of association with chronological age in this study does not necessarily imply that these markers are not age-related at all.

From the clinical perspective, aging biomarkers that would be predictive for chemotherapy-associated adverse events (toxicity, unplanned readmissions), would be highly relevant. However, we found that none of the biomarkers tested was associated with grade II-III-IV toxicity or unplanned readmissions, and neither were the clinical frailty scores (Balducci/LOFS)

Due to the non-randomized design of the study, we cannot exclude some selection bias. The CG was in fact slightly less fit than the CTG at the start of the study, as apparent from LOFS and biomarkers at baseline and by long-term frequency of hospitalization events (between T1 and T2). This was not unexpected, since not only patients at low risk for cancer recurrence, but also patients too frail for chemotherapy, were included as controls. However, this does not influence our conclusions, as we do not compare absolute values of test results at a specific time point, but rather consider differences in evolution over time of clinical and biological aging markers between the groups.

Taken together, we conclude that chemotherapy, after 1 year, does not significantly influence clinical aging parameters, nor does it induce an altered evolution in the most robust aging biomarkers recognized to date (i.e. LTL and IL-6). Nevertheless, other aging biomarkers (MCP-1, TNF-α, IL-10 and IGF-1) evaluated in this study indicated a (mild) potential aging promoting effect of chemotherapy. We found, however, no evidence that changes in these circulating molecules, as a consequence of chemotherapy, do result in clinically relevant changes in frailty, in morbidity, or in higher (all-cause) mortality.

Our study is the first to report a prospective comparison of exclusively older breast cancer patients receiving or not receiving post-operative chemotherapy by measuring several different clinical (GA) and biological aging markers. The results demonstrate that although some biological markers do change during and after chemotherapy, there is no convincing evidence of a clinically relevant acceleration of the aging process. This is an important finding because it emphasizes that chemotherapy should not be denied to older breast cancer patients solely because of their advanced age.

## PATIENTS AND METHODS

### Patient population and clinical assessment

This prospective, multicentre, non-interventional study accrued patients in 2 academic and 3 regional hospitals in Belgium from 2009 until 2012 (www.clinicaltrials.gov/ NCT00849758). Eligible patients for the chemotherapy group (CTG) were female, ≥70 years old with early invasive breast cancer for whom adjuvant chemotherapy was planned according to established risk factors and international guidelines [48]. The scheduled therapy consisted of docetaxel at a dose of 75 mg/m^2^and cyclophosphamide at 600 mg/m^2^every 3 weeks for a total of 4 cycles (TC scheme)[41–43]. Primary prophylaxis with G-CSF (granulocyte-colony stimulating factor) was administered as per standard practice guidelines. In parallel, we enrolled a control group (CG) consisting of early breast cancer patients ≥70 years old for whom chemotherapy was not indicated (or indicated, but judged not to be feasible), and who were administered an aromatase inhibitor as sole adjuvant systemic therapy. Patients either or not received adjuvant radiotherapy according to institution policy. In the chemotherapy group, patients with hormone sensitive tumors also received an endocrine therapy after completion of chemotherapy. Trastuzumab was associated to the adjuvant chemotherapy if the tumor was HER2 positive.

The study was approved by the Ethical Committee of the participating hospitals and written informed consent was obtained from all patients.

Patients were enrolled after surgery. They underwent blood sampling, geriatric assessment (GA) and Quality of Life (QoL) evaluation at three time points. The first time point was between 3 and 6 weeks after surgery, and always before the first chemotherapy administration. The second time point was approximately 3 months after inclusion (day of last chemotherapy), and the last time point was around 1 year after inclusion.

We performed a G8 [49]_ENREF_44 screening test [49] at baseline, and a GA, at each time point. Social data (age, living situation, marital status and educational level) were assessed. Functional status was measured by Katz's Activities of Daily Living (ADL) and by Lawton's instrumental Activities of Daily Living (iADL) scales. A fall history (number of falls during the previous 12 months and presence of fall-related injury) was recorded. We determined cognitive status with the Mini Mental State Examination (MMSE) and mood with the 15-item Geriatric Depression Scale (GDS-15). The nutritional status was assessed using the Mini Nutritional Assessment-Short Form (MNA-SF). Polypathology and severity of medical problems were measured with the Charlson Comorbidity Index (CCI). Geriatric scales have been described in detail by Kenis C. et al [50]. GA results were categorized into “fit”, “vulnerable” and “frail” groups according to Balducci [51, 52]. However, as this categorisation has limitations (e.g. age above 85 is always considered “frail”), we developed a new scoring system, the *Leuven Oncogeriatric Frailty Score* (LOFS), to summarize GA results in a more refined linear score ranging from 10 (very fit) to 0 (very frail). Details are described in appendix 1, and in one of our previous publications [4].

Classical oncological parameters such as Eastern Cooperative Oncology Group - Performance Status (ECOG-PS), tumor characteristics (i.e. tumor subtype according St-Gallen criteria [53] and TNM) and treatment details were recorded. Adverse Events according to CTCAE v4.0 and unplanned readmissions were recorded. An unplanned readmission was identified as a subsequent or repeat hospitalization, which could not have been foreseen at the time of baseline time point [54]. Polypharmacy was assessed by the number of different registered drugs (www.bcfi.be) the patient had been taking during the week preceding inclusion. QoL was assessed with the EORTC QLQ-C30 questionnaire, from which the last two questions (question 29 and 30) were further used to determine ‘global QoL’.

### Blood sampling and measurement of aging biomarkers

At each time point, blood was sampled in 4-mL EDTA K2E tubes for plasma isolation and leukocyte DNA extraction.

Mean leukocyte telomere length (LTL) was measured on leukocyte DNA by qPCR [44] and plasma levels of IL-6, IL-10, IGF-1, TNF-α, MCP-1, and RANTES were assessed by ELISA. Detailed procedures are described in appendix 2.

### Statistics and Endpoints

Statistics and endpoints are described in appendix 3.

## SUPPLEMENTARY MATERIALS


