# Supplementary material for: The impact of adjuvant chemotherapy in older breast cancer patients on clinical and biological aging parameters
Source: Oncotarget. 2016 Apr 18;7(21):29977–88. doi: 10.18632/oncotarget.8796 (PMC5058657; doi:10.18632/oncotarget.8796)
Supplement: Supplementary file 1 [file oncotarget-07-29977-s001.pdf]

The impact of adjuvant chemotherapy in older breast cancer patients on clinical and biological aging parameters

Supplementary Material

Appendix 1

Leuven Oncogeriatric Frailty Score (LOFS)

	LOFS +2	LOFS +1	LOFS +0
ADL	6	5 4	≤ 3
iADL	8	7 4	≤ 3
MMSE	30 28	27 24	≤ 23
MNA-SF	14 12	11 8	≤ 7
CCI	0	1	≥ 2

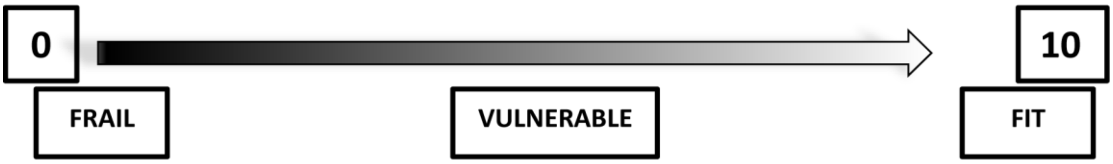

LOFS is a semi-continuous frailty score which integrates results from Activities of Daily Living (ADL), instrumental activities of daily living (iADL), Mini Mental State Examination (MMSE), Mini Nutritional Assessment (MNA-SF) and Charlson Comorbidity Index (CCI). The scoring range for each separate test is separated in three groups, the lowest part (worst score range for each particular test) resulting in a LOFS +0 (no contribution to the final 10-points score), the middle part in +1 (contribution of 1 point), and the highest part in +2 (contribution of 2 points). Subscores from the 5 tests are added up to result in a total score on a scale that ranges from 0 (poorest score; extreme frailty) to 10 (best score, fit patient). Individual results from the LOFS should be interpreted as a gradation of severity in the spectrum of frailty between the two extremes.

Appendix 2 : Methods

Mean leukocyte telomere length (T/S ratio)

Mean leukocyte telomere length was measured on DNA of leukocytes extracted from the buffy coat remaining in the 4 ml EDTA tube after plasma removal. Every DNA sample was first tested for DNA fragmentation by electrophoresis on a 1% agarose gel. Fragmented DNA samples were excluded from further analysis. Telomere Length was assessed by qPCR. According to this method, the relative amount of telomeric DNA (T/S ratio) is calculated based on the Cp values obtained for telomeric DNA (T) and for the single-copy housekeeping gene 36B4 (S), measured in the same sample. All samples were assayed twice in independent qPCR runs, each time in triplicate wells. Each run included a dilution series (i.e. 80, 20, 5 and 1.25 ng) of standard DNA (Human Genomic DNA, Roche cat. no. 11691112001). The T/S ratio for an experimental sample is the amount (ng) of standard DNA that matches the experimental sample for copy number of the telomere template (T), divided by the amount (ng) of standard DNA that matches the experimental sample for copy number of the single-copy gene (S). Primer pairs used were 5'-ACACTAAGGTTTGGGTTTGGGTTTGGGTTTGGGTTAGTGT-3' and 5'-TGTTAGGTATCCCTATCCCTATCCCTATCCCTATCCCTAACA-3' for telomeres and 5'-CAGCAAGTGGGAAGGTGTAATCC-3' and 5'-CCCATTCTATCATCAACGGGTACAA-3' for 36B4. The reaction mixture included 1x LightCycler 480 SYBR Green I Master, telomere primers at 0.6 µM each or 36B4 primers at 0.5 µM each, and 20 ng of template DNA in a total volume of 20 µL. Plates were run on a Roche LightCycler 480 platform, using the following thermal cycling program : activation for 10 min at 95°C; two initiation cycles of 15s at 95°C followed by 15s at 49°C; 35 amplification cycles of 15s at 95°C, 10s at 60°C and 15s at 72°C. Melting curves were then established in order to check amplicon purity.

Measurements of molecules in plasma

Plasmatic levels of cytokines, chemokines and IGF-1 were analyzed by ELISA method following manufacturers' instructions.

IL-6 , IL-10, TNF-alfa, CCL5/RANTES, CCL2/MCP-1, and IGF-1 levels were measured with Quantikine ELISA kit (R&D Systems)

Read-out was performed by dual spectrophotometric measurement: absorbance measured at 570 nm was subtracted from absorbance measured at 450 nm for IL-6, RANTES, MCP-1 and IGF-1. For TNF-alfa and IL-10, absorbance measured at 690 nm was substracted for absorbance measured at 490 nm. All samples were assayed in duplicate. On each microplate, a standard curve, obtained from dilution of a standard with known concentration, was included. From these standard curves concentrations of samples were calculated by a logistic curve-fitting algorithm.

Appendix 3 : Statistics and Endpoints

Statistics

For the primary endpoint, a linear model for longitudinal data was used with telomere length as response variable and time, study arm and their interaction as explanatory variables. An unstructured residual covariance matrix was modelled to account for clustering by repeated measures. For the secondary endpoints, linear models for longitudinal data were used for continuous responses, analogous to primary endpoint analysis. Analyses were performed on transformed responses where needed to improve symmetry of the distribution. Proportional odds models were used for ordinal responses, and logistic regression models for binary outcomes, both with random intercept to account for clustering by repeated measures. Spearman correlations were used for studying the association of aging markers with continuous variables. Kruskal-Wallis tests were performed to compare biomarker levels between more than 2 groups, and Mann-Whitney U tests were used for comparisons between two groups. All tests are two sided, and a 5% significance level is considered for all tests. All analyses have been performed using SAS software, version 9.3 of the SAS System for Windows. Copyright © 2002 SAS Institute Inc. SAS and all other SAS Institute Inc. product or service names are registered trademarks or trademarks of SAS Institute Inc., Cary, NC, USA.

Endpoints

The primary endpoint was to assess whether adjuvant chemotherapy for breast cancer induces accelerated telomere attrition. As secondary endpoints, we examined the impact of chemotherapy on plasma levels of IL-6, IL-10, IGF-1, TNF-α, MCP-1, and RANTES, on GA parameters, clinical frailty scores (LOFS and Balducci) and on QoL. Additionally, we investigated correlations of biological aging markers at inclusion with chronological age and clinical frailty.

Lastly, we investigated whether biological aging markers and clinical aging, at inclusion, were predictive for chemotherapy induced grade II-III-IV toxicity or unplanned readmissions.
